# How are mental simulations updated across sentences?

**DOI:** 10.3758/s13421-019-00928-2

**Published:** 2019-04-08

**Authors:** Lara N. Hoeben Mannaert, Katinka Dijkstra, Rolf A. Zwaan

**Affiliations:** Erasmus University Rotterdam, Burgemeester Oudlaan 50, Mandeville Building, PO Box 1738, 3000 DR Rotterdam, The Netherlands

**Keywords:** Grounded cognition, Mental representation, Situation models, Language comprehension, Discourse, Updating

## Abstract

We examined how grounded mental simulations are updated when there is an implied change of shape, over the course of two (Experiment 1) and four (Experiment 2) sentences. In each preregistered experiment, 84 psychology students completed a sentence–picture verification task in which they judged as quickly and accurately as possible whether the pictured object was mentioned in the previous sentence. Participants had significantly higher accuracy scores and significantly shorter response times when pictures matched the shape implied by the previous sentence than when pictures mismatched the implied shape. These findings suggest that during language comprehension, mental simulations can be actively updated to reflect new incoming information.

Imagine you are reading a story about an eagle that is flying in the sky. The eagle continues to soar for a while before eventually landing in its nest and going to sleep. If you were to make a drawing of the eagle’s final shape, would the wings of the eagle be folded? Anyone with some knowledge of how birds rest in a nest would know that the answer to this question is “yes,” but would not have forgotten the bird’s initial shape. According to the perceptual symbol systems theory (Barsalou, 1999), during language comprehension we activate modal symbols that can be combined in a mental simulation, which may involve the same neural structures as would be used if we were to see the described event in real life. According to Sato, Schafer, and Bergen (2013), these mental simulations are formed incrementally, suggesting that while reading this story about an eagle, we initially created a mental simulation of the eagle having spread wings as it was flying in the air, followed by the inclusion of an eagle with folded wings in the simulation later on. What remains unclear is whether or not the final object replaced the initial simulation, or whether both object states remain activated in that simulation.

Although there is still some debate as to whether mental representations require sensorimotor input or whether amodal symbols are required (e.g., Mahon & Caramazza, 2008), many cognitive psychologists believe that sensorimotor input is required in some form to support language comprehension (Barsalou, 1999, 2008). Indeed, many behavioral experiments have shown that mental simulations can include an object’s shape (Zwaan, Stanfield, & Yaxley, 2002), color (Hoeben Mannaert, Dijkstra, & Zwaan, 2017; Zwaan & Pecher, 2012), orientation (Stanfield & Zwaan, 2001), size (De Koning, Wassenburg, Bos, & Van der Schoot, 2017), and movement during language comprehension (Gentilucci, Benuzzi, Bertolani, Daprati, & Gangitano, 2000; Glenberg & Kaschak, 2002).

In the past, it has been difficult to tease apart whether the match effects found in word–picture or sentence–picture verification tasks are due to the visual system being used, or whether it is only the conceptual system being recruited for the task (Ostarek & Huettig, 2017). However, recent studies using a technique called continuous flash suppression in word–picture verification tasks, where the picture shown is rendered practically invisible by disrupting the processing of visual stimuli (Lupyan & Ward, 2013; Ostarek & Huettig, 2017), have provided evidence that spoken words also activate low-level visual representations. These findings provide support for the idea that conceptual representations also involve the visual system. Moreover, many neuroimaging studies have also illustrated that modality-specific sensory, motor, and affective systems are involved during language comprehension (Binder & Desai, 2011; Hauk, Johnsrude, & Pulvermüller, 2004; Sakreida et al., 2013; Simmons et al., 2007), illustrating that both behavioral and neuroimaging studies support the idea that mental simulations involve sensorimotor activation.

Many studies have targeted the question of *what* is represented in a mental simulation, but more and more researchers are now focusing on *how* mental simulations unfold across texts and their relevance for language comprehension. For instance, a study by Kaup, Lüdtke, and Zwaan (2006) illustrated that responses to pictures that matched the situation described in a preceding sentence were facilitated when the sentences were affirmative (e.g. *The umbrella was open*), but only after a 750-ms delay. This facilitation was no longer present at 1,500 ms, suggesting that the representation may have deactivated at that point in time. Sentences described negatively (e.g. *The umbrella was not closed*), however, only led to facilitation after a 1,500 ms delay, but not after 750 ms. These findings provide evidence for the idea that mental simulations require additional processing time if sentences are complex, and that these simulations can become deactivated after a period of time.

Interestingly, it has also been shown that these simulations can be reactivated at a later point in time if the context requires it. For example, reading sentences implying a certain object shape or orientation leads to faster responses in an object-verification task performed after a 45 minute delay, illustrating that a mental simulation formed during the reading of a sentence can be reactivated if necessary at a later point in time (Pecher, Van Dantzig, Zwaan, & Zeelenberg, 2009). Furthermore, several studies examining the influence of imperfective and perfect verb aspect have shown that mental simulations remain activated longer when there is a description of an ongoing situation (e.g., *The boy was building a doghouse*) compared with a description of a situation that has already occurred (e.g., *The boy had built a doghouse*; Madden & Therriault, 2009; Madden & Zwaan, 2003; Magliano & Schleich, 2000). These studies support the idea that grammatical markers provide cues for how a situation model should be constructed and updated.

Radvansky and Zacks (2011; see also Zwaan & Madden, 2004) explain that this updating process can take various forms: firstly, a new situation model can be constructed (*model creation*); secondly, if new information is consistent with the current situation, it can be incorporated into the existing model (*model elaboration*); thirdly, the model can be altered to accommodate new information (*model transformation*); and finally, it can merge two models into one (*model blending*). Model creation may be used when there is a shift to a new event in a narrative—for instance, when a character moves from one location to the next (Radvansky, 2012). Model transformation occurs when, for example, contradictory information needs to be integrated into the existing situation model. For example, if a person claims to be a vegetarian, but subsequently eats a hamburger (cf. Albrecht & O’Brien, 1993), this would require the model to be transformed to accommodate this contradiction. Model elaboration is what occurs when more information about the current situation is added without requiring a structural change (e.g., reading about a jogger in a marathon and eventually noticing that his shoes are blue. Model blending happens when initially two events are perceived to be distinct, but eventually are considered to be part of the same event. For example, if a man walks into another room to pick up his coat, it may initially be perceived as an event boundary—and thus a separate situation model would need to be constructed. However, once he returns to the room he came from while putting on the coat, it becomes clear that the grabbing and wearing of the coat is part of an ongoing event, and thus the events need to be blended into one coherent situation model.

Given that there are various ways in which a situation model can be updated, how would mental simulations be affected during language comprehension? If mental simulations are simply “the reenactment of perceptual, motor, and introspective states acquired during experience with the world, body, and mind” (Barsalou, 2008, p. 618), then would the process of updating a situation model lead to the activation of multiple states in a mental simulation, or would only the “final” state stay activated? If situation models are representations of the text that underpin language comprehension, then any changes occurring in a mental simulation—an important subcomponent of the situation model—would influence the construction of the situation model. In other words, if mental simulations are directly involved in the comprehension process, then we would expect mental simulations to update dynamically as a narrative unfolds. However, if mental simulations are not directly involved, but are rather a function that activates all relevant perceptual input—which is subsequently distinguished by higher order cognitive processes—then it would make sense for all relevant perceptual input to be activated in a mental simulation. As such, in order to gain a full understanding of how mental simulations are involved in language comprehension, it is important to find out how the updating process affects mental simulations. Most studies have only focused on situation model updating by looking at the slowdown of reading as a measure for this updating process, but these studies fail to provide a complete picture of this updating process. When reading about inflating a balloon, it is of course interesting to know that there is an increase in processing time necessary to comprehend the changes to the situation model, but what exactly happens to the representations described by that sentence? Does the initial shape of the balloon (i.e., deflated) stay activated as the final shape (i.e., inflated balloon) also activates? Or does the deflated balloon representation deactivate as the inflated balloon activates? An fMRI study by Hindy, Solomon, Altmann, and Thompson-Schill (2015) suggests that the brain does encode object state changes, using short sentence items such as “inflate the balloon” to investigate this. Their results suggested that the ventral visual cortex encodes both the initial object state (e.g., deflated balloon) and the final object state (e.g., inflated balloon). Furthermore, the authors suggest that the posterior parietal cortex may be recruited for conceptual binding, so that the distinct states are bound together in a stable representation. These findings suggest that we have to know what an initial object state is in order to comprehend that a change in state is occurring. Possibly, this could mean that when a change in an object state is described, that both object representations are activated in a mental simulation.

To our knowledge, only one study thus far has explored what happens to mental simulations of changing object states. Sato et al. (2013) were interested in finding out whether mental simulations are formed incrementally (i.e., while reading a sentence) or after all the information has been obtained (i.e., at the end of the sentence). If a mental simulation is only formed once all the information has been collected, then it would be unlikely for an initial object state to be activated. If a mental simulation is formed incrementally, however, multiple object states could become activated and potentially deactivated during the process of comprehension. To explore this, Sato et al. had participants read a Japanese sentence where an expectation of an object shape was created, but contradicted at the end of the sentence. For example, in one item, participants read about a person wearing a *yukata* (a cotton kimono) to a fireworks festival, but the *yukata* had been torn apart. As Japanese is a verb-final language, this was an ideal medium by which to create an expectation of an object shape (i.e., a whole *yukata*) and examine what a subsequent contradiction would do to the mental simulation of this event. In order to test which shape would be simulated, they used a picture-verification task either before or after the final verb in the sentence. Their results showed that participants responded faster to the picture matching the shape implied both before and after the final verb, suggesting that mental simulations are formed incrementally and that an initial shape can be deactivated in a mental simulation if a person is presented with information that contradicts earlier expectations.

What remains unclear, however, from Sato et al.’s (2013) findings, is whether an initial shape activation would become deactivated in a mental simulation when no contradictory information is supplied. For example, when reading a sentence pair such as “The eagle was moving through the air. That evening, the eagle was resting in its nest,” the latter sentence does not contradict the event that took place earlier on. Instead, the eagle has spread wings while it is flying, which changes to an eagle having folded wings once it is resting later on. At the end of this sentence pair, would a person still have the initial object state active in a mental simulation, or would this have been deactivated? We are interested in finding out whether implied changes in shape lead to the simultaneous activation of both object shapes, or whether only the final shape remains active in the mental simulation. As most studies examining situation model updating use reading times as a dependent variable, using a sentence–picture verification task offers a unique way to explore how these mental simulations unfold, and whether this updating process requires both visual representations to be active simultaneously, or whether the final representation becomes active as the prior one deactivates. Such a task allows us to glean additional information regarding the nature of mental simulation updating, which cannot be done with reading-time tasks alone.

## The present study

Two experiments were conducted to examine how mental simulations are updated when changes in implied shape are described over the course of several sentences, using a sentence–picture verification task. Participants had to read either two (Experiment 1) or four (Experiment 2) sentences that described either a change in shape (change condition) or no change in shape (constant condition), followed by a picture that either matched or mismatched the shape implied by the final sentence, where they had to decide whether the pictured object was mentioned in the text.

Given that previously simulated information can be reactivated when necessary (Pecher et al., 2009; Sundermeier, van der Broek, & Zwaan, 2005) and can continue to influence language comprehension in the future (O’Brien, Cook, & Guéraud, 2010), it is possible that, in a context where both object states are implied immediately one after the other, both would remain activated in order to update the situation model. This could potentially occur during model elaboration as opposed to model creation (Radvansky & Zacks, 2011). It is also possible, however, that due to mental simulations being formed incrementally, that the initial object state is replaced immediately when the second state is mentioned. This explanation would fall in line with what is proposed in the event horizon model (Radvansky, 2012). According to the event horizon model, a new model is created at event boundaries and could therefore suggest the initial object state is replaced immediately. If the newer object state is then activated in the new situation model, then response times should be shorter when the new object is displayed compared with when the first object is displayed. As such, if reading about a change in an object’s shape is considered to be an event boundary, then we can expect shorter response times when the picture shown matches the changed object’s shape compared to when it mismatches this shape. If instead the model is merely elaborated upon, then no differences would be expected when the picture shown matches the first-mentioned or last-mentioned object.

We hypothesized that in Experiment 1, where one shape is mentioned directly after the other, that both shapes would remain activated in the mental simulation as we expected model elaboration to occur, rather than creation. Therefore, we predicted to find no significant differences in the response times between the match and mismatch condition in the shape-change condition. We did expect a match effect in the shape-constant condition, as this would be consistent with findings from previous studies (e.g., Zwaan & Pecher, 2012; Zwaan et al., 2001). In Experiment 2, however, the final object shape was emphasized using the final three sentences (out of four total). In this experiment, we expected that the initial shape would become deactivated as more emphasis was placed on the final shape. Specifically, we predicted a significant match advantage in the response times in both the shape-change and shape-constant conditions in this experiment. We made no specific predictions regarding the accuracy scores in both Experiments 1 and 2.

### Ethics statement

The participation in all experiments and in the norming study was voluntary. The participants subscribed to the experiments online via the university platform and were told that by signing up for a study, they declare to voluntarily participate in this study. They were briefed with the content of each study, but obtaining further written consent was not required by the Ethics Committee of Psychology at the Erasmus University Rotterdam, The Netherlands, who approved the project, because the experiments were noninvasive and the data collected were processed anonymously.

### Preregistration

The predictions, exclusion criteria, design, methods, analyses, and materials of all the experiments reported in this article were preregistered in advance of data collection and analysis on the Open Science Framework (OSF) to ensure confirmatory procedures were conducted according to a priori criteria.^1^ Analyses that were not preregistered are referred to in this article under the heading Exploratory Analyses.

## Experiment 1

### Method

#### Norming study

In the current study, participants read sentences where a certain shape is implied, rather than explicitly stated. In order to ensure that the sentences did imply the shape we intended them to, a norming study was conducted. Forty-one participants were recruited via Mechanical Turk (www.mturk.com) and were paid $3.50 for completion of the survey, which took approximately 30 minutes to complete. The participants had a mean age of 35.10 years (*SD* = 10.87) and consisted of 19 females and 22 males.

Participants in the norming study read 280 sentences (seven sentences were written for 40 different items for Experiments 1 and 2) that implied a certain object shape (e.g., *The bat was entering the cave*) and had to determine which of the shown pictures best matched the sentence. There was also an option where participants could state that neither picture matched the sentence they read and they could provide comments to elaborate on their reasoning. By doing the norming study in this manner, we could ensure that the final items we would end up using would actually imply the shape we wanted it imply. For example, for the item about an eagle, 91.30% of participants agreed that our matching picture corresponded to the shape implied by the sentence “The eagle was moving through the air,” while 82.61% agreed that our matching picture corresponded to the sentence “The eagle was resting in its nest.” If we consider an item to be the object of the sentences, then each item would lead to seven ratings per participant (i.e., one rating for the sentence about the eagle moving through the air, another rating for the sentence about the eagle resting in the nest, and so on). For the results, we looked at the sentence of an item that contained the lowest percentage of agreement across participants in order to be conservative in our final selection of stimuli. The results of the study illustrated that the stimulus set contained several items where the minimum average agreement on what shape matched which sentence was less than 61%. Furthermore, on some of the items, participants made comments suggesting the item contained an incorrect logical match between sentence and picture. To improve the quality of the stimulus set and to ensure the final set contained a number of stimuli that was a multiple of four (due to counterbalancing), 12 items were removed. This left us with 28 experimental items that had a minimum average agreement of 77.52% (*SD* = 7.73%) regarding which picture best represented which sentence.

#### Participants

We expected to need 84 participants to find an effect if it existed—based on a power analysis performed on the results of the Zwaan et al. (2002) study—and therefore continued data collection until this goal had been met, replacing participants that had to be excluded due to having total accuracy scores below 80%. As a result of this measure, only one participant was excluded and replaced. The final sample consisted of 84 participants (ages 17–29 years, *M*_age_ = 20.33 years, *SD*_age_ = 2.04 years, 71 females, 13 males), who were students of the International Bachelor Programme of Psychology (IBP) at the Erasmus University Rotterdam in The Netherlands. IBP students are required to have a minimum level of 80 on the Test of English as a Foreign Language (TOEFL), or a 6.0 on the International English Language Testing System (IELTS), or be native speakers of English. Participants were reimbursed with course credit.

#### Sentences

**Fig. 1:**
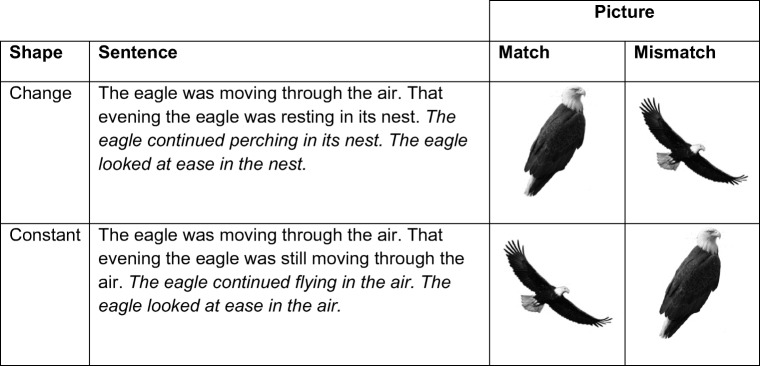
Example of stimulus material used in Experiments 1 and 2 (italics). Experiment 2 used the first sentences from Experiment 1 and had two sentences added at the end to continue emphasizing one shape. Pictures under “Match” refer to a picture matching the shape implied by the final sentence, whereas “Mismatch” refers to a picture mismatching the shape implied by the final sentence. The full list of stimuli can be viewed here: https://mfr.osf.io/render?url=https://osf.io/3hrq8/?action=download%26mode=render

Twenty-eight experimental sentence pairs and 28 filler sentence pairs were used in the experiment for each experiment block. The sentence pairs in one block involved an implied change in shape (change condition), and the sentences in the other block involved no change in shape (constant condition). See Fig. 1 for an example of the sentence stimuli. Of the experimental sentence pairs, two versions were created for counterbalancing purposes to account for typicality effects. The first version ensured that the final sentence implied one of two possible object shapes (e.g., eagle–folded wings), while the second version ensured the final sentence implied the other possible shape (e.g., eagle–spread wings). To be able to counterbalance in this way without manipulating time aspect in the sentences, we could only use objects that were reversible in shape. For example, an eagle is able to fold its wings and then spread them again, and vice versa. An egg, however, could only ever start out as a whole egg and then become a fried egg, not the other way around.

#### Pictures

Eighty-four pictures were created for use in this experiment, of which 56 were used for the experimental sentences and 28 for the filler sentences. During the experimental procedure, each sentence pair was followed by a picture that either matched or mismatched the implied shape, thus requiring the creation of two picture versions of a particular object. For example, if the sentence stated that the eagle was in the air, then a picture of either an eagle with spread wings or a picture of an eagle with folded wings could be shown. The pictures were obtained from the Internet (Google Image search engine) and were edited with the Paint.NET software to be displayed as grayscale (to ensure that effects of color could not confound the results), to not exceed a 300 × 300 pixel resolution (approximately 7.9 cm × 7.9 cm on-screen). The experiment was programmed using E-Prime 2.0 Professional, and participants completed the experiments in isolated cubicles with computers equipped with 24.1-in. TFT-IPS screens, with a resolution of 1,920 × 1,200 and a ratio of 16:10.

#### Design and procedure

The experiment was a 2 (match: match vs. mismatch) × 2 (shape: change vs. constant) × 2 (block: Block 1 shown first vs. Block 2 shown first) × 2 (sentence: Version 1 vs. Version 2) mixed-subjects design, resulting in eight counterbalanced lists. *Match* and *shape* were tested within subjects, meaning that participants viewed pictures that matched and mismatched implied shape, and viewed sentences that either implied a change in shape (change condition) or did not (constant condition). *Block* and *sentence* were between-subjects variables that ensured that, firstly, half of all participants were shown Block 1 during Session 1 (containing only the change-condition items) and then Block 2 during Session 2, while the other half were shown Block 2 during Session 1, followed by Block 1 during Session 2. Participants completed each session 1 week apart to ensure that there were no carryover effects from one set of materials to another. Secondly, each experimental sentence pair could imply one of two object states (i.e., a sentence implying an eagle with spread wings or a sentence implying eagle with folded wings). This variable was also tested between-subjects so that half of all participants viewed the sentence implying one of the object states and that the other half viewed the sentence implying the other object state. Within the same block, participants received each item once. We counterbalanced the experiment in this way to ensure that neither block order nor problems with typicality could be possible explanations for the results.

Participants were instructed that they would perform a self-paced reading task using the spacebar and that they would see a picture after the second sentence, and that they must answer whether the pictured object was mentioned in the previous sentence. They were instructed to press the *L* key for YES responses and the *A* key for NO responses. Participants first received five practice sentences. A trial proceeded as follows: the > sign was shown for 1,000 ms in the center of the screen (left aligned), signifying that they would receive a new sentence sequence. Following this, they saw the first sentence (left aligned), and had to press space to signify they understood the sentence, before immediately seeing the second sentence on the screen. Once they pressed the spacebar, they saw a fixation cross in the center (center aligned) of the screen for 500 ms to prepare them for a response. We chose 500 ms as the interstimulus interval as this is commonly used in sentence–picture verification studies (e.g., De Koning et al., 2017; Hoeben Mannaert et al., 2017; Zwaan & Pecher, 2012). Following the fixation cross, they saw the picture in the center of the screen, which remained on-screen until they had given a response. All of the experimental items (i.e., both the match and mismatch conditions) required a YES response, whereas all of the filler items required a NO response. Half of all filler items also ended with a comprehension question, to ensure they properly read the sentences, where they had to give a YES/NO response using the *L* and *A* keys, respectively.

#### Data analysis

A repeated-measures analysis of variance (rmANOVA) was conducted to test whether participants were faster in their response and whether they were more accurate when the picture matched than when it mismatched for both the change and constant conditions. Only the response times for accurate responses were used in the final analysis, and the median reaction times were analyzed instead of the mean response times. Many experiments using the sentence–picture verification task use the median for the analyses, as it eliminates the necessity to make decisions on outliers (such as use of cutoffs based on standard deviations, absolute RTs, or other methods; Stanfield & Zwaan, 2001; Zwaan & Pecher, 2012). Subject analyses are shown with the subscript “1” (e.g., *F*_1_), and item analyses^2^ are shown with the subscript “2” (e.g., *F*_2_).

### Results

#### Accuracy

Table 1 shows a summary of the descriptives for the change and constant conditions. The rmANOVA for accuracy demonstrated a main effect for match, where participants in the match condition responded more accurately than in the mismatch condition, *F*_1_(1, 83) = 17.63, *p* < .001, η_p_^2^ = 0.175; *F*_2_(1, 27) = 15.79, *p* < .001).^3^ A main effect for shape was also found, where participants in the shape-change condition responded more accurately than participants in the shape-constant condition, *F*_1_(1, 83) = 8.71, *p* = .004, η_p_^2^ = 0.095; *F*_2_(1, 27) = 4.24, *p* = .049. There was no significant interaction between match and shape, *F*_1_(1, 83) = 0.003, *p* = .959; *F*_2_(1, 27) = 0.21, *p* = .648. Paired-sample *t* tests showed that there was a significant match effect in both the shape-change, *t*_1_(83) = 3.59, *p* < .001, and the shape-constant conditions, *t*_1_(83) = 2.18, *p* = .032. The item analyses showed no significant effect of picture version, *F*_2_(1, 27) = 0.06, *p* = .809.

**Table 1:** Overview of match effects in Experiments 1 and 2

	Accuracy		Response time			
	Match *M* (*SD*)	Mismatch *M* (*SD*)	Match *M* (*SD*)	Mismatch *M* (*SD*)	Effect size (Cohen’s *d*_*z*_)	*BF*_*10*_
Experiment 1						
Change	0.97 (0.05)	0.95 (0.07)	572.90 (150.20)	607.50 (224.50)	-0.27	4.52
Constant	0.95 (0.09)	0.92 (0.08)	588.60 (181.30)	614.80 (181.50)	-0.22	1.62
Experiment 2						
Change	0.97 (0.05)	0.95 (0.06)	655.00 (233.60)	679.60 (268.40)	-0.22	1.53
Constant	0.97 (0.05)	0.91 (0.14)	646.60 (227.90)	662.40 (264.80)	-0.13	0.43

*Note.* Response times are shown in ms. Reported effect sizes are for the comparison of response time. Bayes factors were calculated using a Cauchy prior of 0.707 as a one-sided Bayesian paired-samples *t* test, using the JASP software (Version 0.9.0.1) for the calculations

#### Response time

The rmANOVA for response time (performed on correct responses only) found a main effect for match, where participants responded significantly faster when the picture matched than when it mismatched the shape implied by the final sentence, *F*_1_(1, 83) = 10.80, *p* = .001, η_p_^2^ = 0.115; *F*_2_(1, 27) = 9.95, *p* = .004.^4^ There was no significant main effect of shape, *F*_1_(1, 83) = 0.46, *p* = .502; *F*_2_(1, 27) = 0.84, *p* = .366, nor a significant interaction between shape and match, *F*_1_(1, 83) = 0.189, *p* = .665; *F*_2_(1, 27) = 0.21, *p* = .654. Paired-sample *t* tests showed that participants were significantly faster in the match condition in both the shape-change, *t*_*1*_(83) = −2.51, *p* = .014, and the shape-constant conditions, *t*_1_(83) = −2.02, *p* = .047. There was also a significant effect of picture version in the item analyses, *F*_2_(1, 27) = 4.72, *p* = .039.

Exploratory analyses: Comprehension accuracy

To examine whether participants did properly read both sentences in the experiment, the accuracy scores to the comprehension questions were analyzed. Sixteen comprehension questions focused on the final sentence in the pair, whereas 12 questions focused on the first sentence in the pair. Average comprehension accuracy was high at 89.07% (*SD* = 12.20%) for questions focused on the first sentence as well as for questions targeted at the second sentence (*M* = 88.69%, *SD* = 11.61%). A paired-samples *t* test found no significant difference between comprehension accuracy for questions targeting Sentence 1 and Sentence 2, *t*(83) = .31, *p* = .761.

### Discussion

As can be seen from Table 1, overall accuracy of participants was high in each condition. However, even though it was high, participants still responded less accurately when the picture mismatched the final object shape in both the change condition (2% difference) and the constant condition (3% difference). The participants’ task was to respond as quickly and accurately as possible whether the pictured object was mentioned in the previous sentence. Even though this was clearly the case, participants still responded in such a way that when the picture mismatched the implied shape, they were slightly less likely to press the YES response. This could suggest that participants are more inclined to match the picture they see to the image that is present in their mental simulation, rather than compare it to the text base. Furthermore, participants also responded slightly more accurately in the shape-change condition compared with the shape-constant condition. The event horizon model (Radvansky, 2012) provides a potential explanation for this effect. According to this model, memory is enhanced during the perception of an event boundary. As such, it could be that the change condition led to the perception of an event boundary but that this was not the case when there was no implied change in shape. However, the fact that these percentage differences are so small limits the overall strength of these conclusions.

We also found a standard match effect for both the shape-change and constant conditions, which went against our expectations. The results suggest that the most recently implied shape is more highly activated than the first-mentioned shape in situations that involve a change in shape. What this suggests is that mental simulations appear to update in a manner that replaces the initially simulated object, rather than activating both objects in unison. This would mean that, although an object can be reactivated when necessary (such as in Pecher et al., 2009), this is not required for the purpose of updating mental simulations.

Additionally, it looks as though the updating process does not seem to take additional cognitive effort during object changes compared with when there is no change occurring, as no significant differences in response times were found between the change and constant conditions. It is also possible, however, that no differences were found between the change and constant conditions because both required model creation, as the sentence items included a time shift, which has been related to slowdown of reading times in past studies (e.g., Speer & Zacks, 2005) and thus could have also influenced response times during object-verification.

## Experiment 2

Experiment 1 illustrated that the final implied object shape is more activated in a mental simulation than the initial one. In Experiment 2, we wanted to see whether this result would replicate using four sentences instead of two. Most studies using the sentence–picture verification paradigm only examine the match effect using one sentence, therefore it is of interest to see whether this effect holds out under more natural conditions. To test this, the same stimuli were used as in the Experiment 1, except that two more sentences were added to increase the time between the activation of the initial shape and the response. Thus, in each item, the first sentence implied one shape of an object, whereas the final three sentences implied either another shape of that object (i.e., in the shape-change condition) or again the same shape (i.e., in the shape-constant condition). We expected to find a match advantage for both the shape-constant and the shape-change conditions in Experiment 2.

### Method

#### Participants

We again aimed to have 84 participants in our sample and therefore continued data collection until this goal had been met, replacing participants that had to be excluded due to having total accuracy scores below 80%. As a result of this measure, seven participants were excluded and replaced. The final sample consisted of 84 participants (ages 18–47 years, *M*_age_ = 21.08 years, *SD*_age_ = 4.32 years, 62 females), who were IBP students at the Erasmus University Rotterdam in The Netherlands, and had a minimum level of 80 on the TOEFL, or a 6.0 on the IELTS. Participants were reimbursed with course credit.

#### Materials

The sentence items contained four sentences. The first two sentences of the item were identical to the ones from Experiment 1. The final two sentences continued implying the shape mentioned in the second sentence. Aside from this change in the stimuli, all materials were the same as that of Experiment 1. An example of the stimuli can be seen in Fig. 1.

#### Design and procedure

The design and procedure of Experiment 2 were the same as that of Experiment 1.

### Results

#### Accuracy

The data from Experiment 2 were analyzed using the same method as in Experiment 1. Table 1 shows a summary of the descriptives for the change and constant conditions. The rmANOVA for accuracy found a main effect for match, where participants in the match condition responded more accurately than in the mismatch condition, *F*_1_(1, 83) = 13.81, *p* < .001, η_p_^2^ = 0.14; *F*_2_(1, 27) = 11.90, *p* = .002).^5^ A main effect for shape was also found in the subject analyses (but not in the item analyses), where participants in the shape-change condition responded more accurately than participants in the shape-constant condition, *F*_1_(1, 83) = 4.09, *p* = .046, η_p_^2^ = 0.05; *F*_2_(1, 27) = 3.57, *p* = .069. There was also a significant interaction between match and shape, *F*_1_(1, 83) = 4.74, *p* = .032, η_p_^2^ = 0.05; *F*_2_(1, 27) = 8.63, *p* = .007. Paired-sample *t* tests showed that the match effect was significant for both the shape-change, *t*_1_(83) = 2.34, *p* = .022, and the shape-constant conditions, *t*_1_(83) = 3.36, *p* = .001. There was no significant effect of picture version, *F*_2_(1, 27) = 0.53, *p* = .473.

#### Response time

**Fig. 2:**
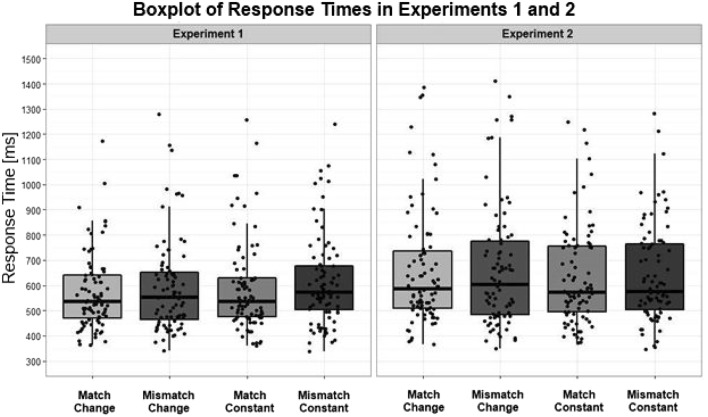
Box plot illustrating a comparison of response times and spread of data for Experiments 1 and 2

The rmANOVA for response time (performed on correct responses only) found a main effect for match in the subject analyses (but not in the item analyses), where participants responded significantly faster when the picture matched than when it mismatched the shape implied by the final sentence, *F*_1_(1, 83) = 5.54, *p* = .021, η_p_^2^ = 0.06; *F*_2_(1, 27) = 2.28, *p* = .143).^6^ There was no significant main effect of shape, *F*_1_(1, 83) = 0.25, *p* = .615; *F*_2_(1, 27) = 0.08, *p* = .776, nor a significant interaction between shape and match, *F*_1_(1, 83) = 0.22, *p* = .638; *F*_2_(1, 27) = 1.10, *p* = .304. Paired-sample *t* tests illustrated no significant match effect for the shape-change condition, *t*_1_(83) = −1.99, *p* = .050, and the shape-constant condition, *t*_1_(83) = −1.21, *p* = .228. Figure 2 illustrates a boxplot comparison of the response times per condition for Experiments 1 and 2. There was also no significant effect of picture version, *F*_2_(1, 27) = 0.09, *p* = .772.

Exploratory analyses: Meta-analysis

**Fig. 3:**
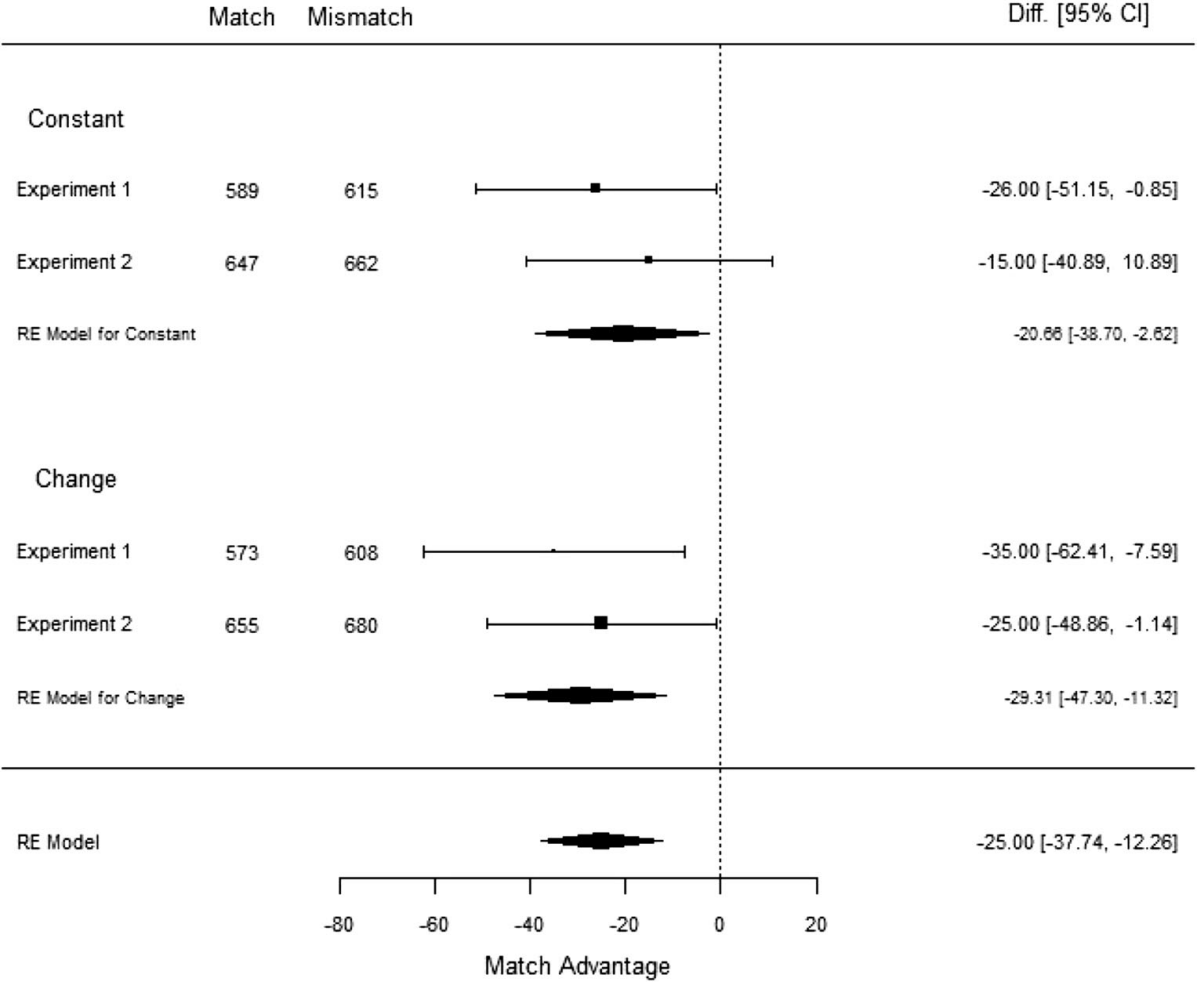
Meta-analysis of Experiments 1 and 2

We performed a meta-analysis on the data to examine the evidence for a match effect existing in the shape-change and shape-constant conditions (see Fig. 3). The meta-analysis was performed using R Version 3.3.2 (R Core Team, 2016) using the package *metafor* (Viechtbauer, 2010). The code can be viewed in the Appendix. The results illustrated a 29.31-ms match advantage in the shape-change condition, and a 20.66-ms match advantage in the shape-constant condition, both significant (*p* = .0014 and *p* = .025, respectively), providing overall support for the notion that comprehenders do actively update their mental simulations.

#### Comprehension accuracy

To examine whether participants did properly read the sentences in the experiment, the accuracy scores to the comprehension questions were analyzed. Sixteen comprehension questions focused on the second sentence, whereas 12 questions focused on the first sentence. Average comprehension accuracy was high at 85.42% (*SD* = 10.97%) for questions focused on the first sentence as well as for questions targeted at the second sentence (*M* = 80.58%, *SD* = 10.72%). A paired-samples *t* test found that participants were significantly more accurate on questions targeting the first sentence compared with the second sentence, *t*(83) = 3.96, *p* < .001.

#### Mismatch accuracy

To further examine the interaction found in the accuracy scores, we performed a paired-samples *t* test to see whether the difference in mismatch accuracy between the shape-constant and shape-change conditions were significant. The results showed that when the picture mismatched the shape implied, participants were significantly more accurate in the shape-change condition (*M* = .95, *SD* = .06) than in the shape-constant condition (*M* = .91, *SD* = .14), *t*(83) = 2.37, *p* = .020.

### Discussion

The aim of Experiment 2 was to find out whether the updating of mental simulations still takes place when participants read four sentences, where the first sentence implies one shape and the final three imply another. The analysis of the accuracy scores revealed an interesting pattern, where participants were most accurate in the match condition, regardless of whether the sentences implied a change in shape or not, but were significantly less accurate in the mismatch condition, which interacted with the shape condition. Inspecting the data more closely, it appears that participants were more accurate in the mismatch condition when a change in shape was implied compared to when no change was implied. It is possible that this finding is due to enhanced memory during event boundaries as proposed by the event horizon model, but then we would have expected to also find better accuracy in match condition when a change in shape was implied, which we did not. An alternative explanation for this finding is that participants in the shape-constant condition experienced a continued reinforcement of a single shape (e.g., an eagle with spread wings) over the course of four sentences. If they are then confronted with a picture that displays a shape that does not overlap with what is present in their mental simulation, they are more likely to state that the pictured object was not mentioned in the previous sentences. In the shape-change condition this would not be the case, as both shapes would have been mentioned in the sentences, resulting in slightly higher accuracy scores. Similar to Experiment 1, however, given that the differences in percentage accuracy is so small between conditions, these conclusions should be interpreted with necessary caution.

The analysis for response times in Experiment 2 did not show the interaction found for accuracy scores. Instead, only a main match effect was found for subjects, where participants were again faster at responding when the picture matched the shape implied by the final sentence than when it mismatched. The paired-samples *t* test, however, illustrated no significant match effect in the shape change and constant conditions. Similarly, the item analyses found no significant match effect. We expect that these findings may be explained by the less natural wording of the sentences in the shape-constant condition. Although we had aimed to create more realistic stimuli by adding more sentences, it is possible that our manipulation caused the opposite effect. The continued emphasis of only one shape throughout all four sentences may have encouraged a surface-level representation, which may have resulted in a weaker activation of all relevant visual representations, leading to a lack of a match effect. Nevertheless, when we conducted a meta-analysis over the data from Experiments 1 and 2, we found a significant match effect of 29.31 ms for the shape-change condition, and a significant match effect of 20.66 ms for the shape-constant condition. This again supports the idea that mental simulations can be actively updated.

Finally, we found that participants were significantly more accurate on the comprehension questions targeting the first sentence compared with those targeting the second sentence. It is possible that this could be explained by the primacy effect, being that items coming first are remembered better than those that come directly afterward.

## General discussion

Over the past decade, much research has been conducted to find out which object properties are represented in mental simulations. Recently, however, more and more researchers have become interested in understanding what the underlying mechanisms of mental simulations are and how they unfold during language comprehension. We were specifically interested in how mental simulations update when a change in shape is implied over the course of two (Experiment 1) and four (Experiment 2) sentences, using a sentence–picture verification task to test this. We hypothesized that if you imply one object shape in one sentence, and then imply the other in the sentence that follows, both shapes would remain active in a mental simulation, leading to no match advantage. We further hypothesized that when more sentences are added that continue to emphasize the final object shape, only the final shape will remain active in a mental simulation, leading to a match advantage.

Our findings do not support our first hypothesis and provide tentative support for our second hypothesis. We found a significant match effect across the shape-change and shape-constant conditions in both Experiments 1 and 2. Upon closer investigation, we found that both the shape-change and shape-constant conditions in Experiment 1 had a significant match effect, but neither condition was significant in Experiment 2. In order to determine whether the most recent shape is more activated in the mental simulation when a change in shape is implied, a meta-analysis was conducted over the data from both experiments. The findings from the meta-analysis support the conclusion that participants have the final shape more activated in the mental simulation, with an overall match advantage of 29.31 ms.

During the process of language comprehension, a comprehensive situation model is built that results in the same (or similar) sensorimotor activation as when the described event is experienced. In a task where participants are asked to compare what they see in the picture with what they have read, it would be possible for them to either compare the picture with the surface structure of the text or to compare it to the constructed situation model. In the first case, we would not expect a difference between any conditions as we tried to avoid making explicit references to object shapes. If the picture is compared at the level of the situation model, however, we would expect the matching picture to lead to faster responses, as the situation model includes all other information that is not only explicitly mentioned in the text (but is still relevant to the aspects of the situation). This is indeed what we found in the current study. If the sentence–picture verification task causes us to compare the viewed picture with our constructed situation model, it would make sense that when there is overlap between the two, we are more likely to answer “yes” in the context of this task. This then also explains why accuracy was higher in the shape-change condition, as the picture will always overlap with one of the implied shapes. In the shape-constant condition, however, there are more constraints on what the object shape could be, so if there is less overlap between picture and model (i.e., in the mismatch condition), it makes sense that there are fewer people who would choose to answer in favor of the picture having been mentioned in the previous sentence.

An alternative explanation to this boosted accuracy effect in the shape-change condition comes from the event horizon model (Radvansky, 2012), which proposes that memory is enhanced at event boundaries. Although we cannot conclude that there were event boundaries in between the sentences of the shape-change condition, as this was not tested, if participants indeed perceive the two different object shapes described in the text as separate events (i.e., model creation; Radvansky & Zacks, 2011), it would explain why there was enhanced accuracy in this condition.

In the introduction, we stated that it makes sense that mental representations can be reactivated at a later point in time if the context requires it (e.g., Pecher et al., 2009; Sundermeier et al., 2005). Indeed, if the comprehender cannot reactivate a previously encountered object state, then an explicit understanding of changes involving that state would become difficult to grasp. However, our results seem to suggest that for the purposes of updating mental simulations, no reactivation of prior object states are necessary.

The results from the current study support the idea that mental simulations are updated when there is an implied change in object shape. At this point we cannot determine whether our results are inconsistent with those reported by Hindy et al. (2015), who concluded in an fMRI study that both the initial object state (e.g., deflated balloon) and the final object state (e.g., inflated balloon) are encoded when an object changes shape in a sentence (e.g., “inflate the balloon”), as it is possible that there would still be some activation of the initial object state left. Our results do support the findings of Sato et al. (2013), who reported that mental simulations can be updated when participants receive information contradictory to the initially simulated shape.

Now that it has been established that mental simulations change the activation levels of the object traces during the process of updating, future research could focus on how much of the initial object trace remains activated when there is new incoming information, which could be investigated by increasing the ISI between the final sentence and the picture. Furthermore, it may prove beneficial to replicate the current study to improve the reliability of these findings.

### Limitations

The current study has a few limitations that may limit the strength of our conclusions. Although the current study used more sentences than are typically used in a sentence–picture verification paradigm with the intent to create more natural discourse, the stimuli used could still be considered impoverished textoids compared with that of common discourse seen in everyday life, which limits the generalizability of our findings (Graesser, Millis, & Zwaan, 1997). Additionally, due to the difficulty in creating items that can both change and reverse shape, it is possible that several items may refer to an object’s shape more explicitly than others, though we did our best to ensure that this was not the case. As such, it would be prudent for future studies to continue trying to improve the quality of the texts used in experiments.

A second potential limitation to this study are the task demands of a sentence–picture verification task, as there is some debate as to whether this paradigm encourages explicit visual processing, and that by extension, outside of this experimental setting, the nature of these representations would vary. Indeed, sentence–picture verification tasks on their own are unlikely to be valid in terms of the conclusions that can be made regarding visual representations. However, there are many studies that have found match effects using various methods, such as electroencephalography (Coppens, Gootjes, & Zwaan, 2012), memory tasks (Pecher et al., 2009), and naming tasks (Zwaan et al., 2002), which all support the idea that the visual system is recruited during language comprehension.

A final limitation to our study is that the comprehension questions we asked the participants were not equally targeting Sentences 1 and 2, and that a significant difference was found in the comprehension accuracies between the two sentences in Experiment 2. Although comprehension accuracy was not a variable of interest to us during the design of this experiment, those results are still relevant, as the significant difference in comprehension accuracy could imply that participants read the first sentence more carefully than the remaining sentences in that item. However, as the number of questions was unbalanced with regard to the sentence’s serial position (i.e., 12 questions targeting Sentence 1 vs. 16 questions targeting Sentence 2), strong conclusions cannot be drawn here. We can conclude that overall comprehension accuracy was high in both Experiments 1 and 2, suggesting that participants read the items. Furthermore, it is unlikely that we would have found the effects we did in the current study if participants had not read the other sentences in each item. Future studies, however, should ensure that comprehension questions equally target the various sentences in an item, so this can be explicitly tested.

We can conclude that mental simulations can be updated after changes in shape are implied across several sentences, and that this most likely occurs through the activation of the final shape and the deactivation of the initial shape. Future research could focus on how much of the initial object trace remains activated when there is a change occurring in a narrative.

## Appendix

The meta-analysis was performed using R 3.3.2 (R Core Team, 2016) using the package *metafor* (Viechtbauer, 2010) with the following code:

## Footnotes

1 The preregistration can be viewed on https://osf.io/f7m3q/register/565fb3678c5e4a66b5582f67

2 The item analyses were not preregistered but were included as part of the main analyses as this is conventional.

3 These analyses excluded the counterbalancing measures such as “sentence version” and “block order” as these were not included in our preregistered analysis plan. When included, the results for match, *F*(1, 76) = 17.59, *p* < .001, and shape, *F*(1, 76) = 9.86, *p* = .002, remained the same. The effect sizes of the counterbalancing measures are not reported due to lack of theoretical relevance (Madden-Lombardi, Dominey, & Ventre-Dominey, 2017; Pollatsek & Well, 1995; Raaijmakers, Schrijnemakers, & Gremmen, 1999).

4 These analyses excluded the counterbalancing measures such as “sentence version” and “block order” as these were not included in our preregistered analysis plan. When included, the results for match, *F*(1,76) = 10.36, *p* = .002, and shape, *F*(1, 76) = .65, *p* = .424, remained the same.

5 These analyses excluded the counterbalancing measures such as “sentence version” and “block order” as these were not included in our preregistered analysis plan. When included, the results for match, *F*(1, 76) = 14.19, *p* < .001, shape, *F*(1, 76) = 4.06, *p* = .047), and the interaction between match and shape, *F*(1, 76) = 4.78, *p* = .032, remained the same.

6 These analyses excluded the counterbalancing measures such as “sentence version” and “block order” as these were not included in our preregistered analysis plan. When included, the results for match, *F*(1, 76) = 4.75, *p* = .032, and shape, *F*(1, 76) = 0.07 *p* = .797, remained the same.
